# Validation of an HPLC-MS/MS method for the quantification of pesticide residues in Rice and assessment of the washing effect

**DOI:** 10.1016/j.fochx.2024.101938

**Published:** 2024-10-29

**Authors:** Filipa Carreiró, Sílvia Cruz Barros, Carla Brites, Ana Rita Mateus, Fernando Ramos, Duarte Torres, Ana Sanches Silva

**Affiliations:** a National Institute for Agrarian and Veterinary Research (INIAV), I.P., Av. da República, 2780-157 Oeiras, Portugal; b University of Coimbra, Faculty of Pharmacy, Polo III, Azinhaga de St. Comba, 3000-548 Coimbra, Portugal; c EPIUnit–Institute of Public Health, University of Porto, 4200-450 Porto, Portugal; d Faculty of Nutrition and Food Sciences, University of Porto, 4200-393 Porto, Portugal; e Laboratory for Integrative and Translational Research in Population Health (ITR), 4200-450 Porto, Portugal; f GREEN-IT Bioresources for Sustainability, ITQB NOVA, Av. da República, 2780-157 Oeiras, Portugal; g Centre for Animal Science Studies (CECA), ICETA, University of Porto, 4501-401 Porto, Portugal; h REQUIMTE/LAVQ, R. D. Manuel II, Apartado, 55142, Porto, Portugal; i Associate Laboratory for Animal and Veterinary Sciences (Al4AnimalS), 1300-477 Lisbon, Portugal

**Keywords:** Rice, Pesticide residues, Retention factors, Washing process, Processing factors, Food processing

## Abstract

A method was validated to determine 121 pesticide residues (carbamates, organophosphates, organochlorines, and pyrethroids) in rice samples, following the guidance document SANTE/11312/2021v2. QuEChERS method was selected for pesticide extraction, and the extract was analyzed by HPLC-MS/MS. The methodology demonstrated precision and accuracy, with recovery rates ranging from 70 % to 119 %. Additionally, the study aimed to assess the effects of washing on residue levels of 121 pesticides in contaminated long-grain rice samples. Subsequently, the rice underwent washing, and the pesticide residues were determined in the samples to evaluate the retention factor. The results suggest that the washing process can enhance the elimination of pesticide residues in rice, around 40 % of pesticides have reduced between 40 and 60 %, and 10 % of pesticides reduced by more than 60 %. This research contributes with valuable insights for improving food safety measures in the context of pesticide-contaminated cereals.

**Chemical compounds:**

(Azoxystrobin) PubChem CID: 3034285

(Cadusafos) PubChem CID: 91752

(Diazinon) PubChem CID: 3017

(Epoxiconazole) PubChem CID: 107901

(Fenamidone) PubChem CID: 10403199

(Fenpropimorph) PubChem CID: 93365

(Mepanipyrim) PubChem CID: 86296

(Pirimiphos-methyl) PubChem CID: 34526

(Propiconazole) PubChem CID: 43234

(Spiroxamine) PubChem CID: 86160

## Highlights

• QuEChERS method was used for pesticide extraction, and HPLC-MS/MS was validated per SANTE/11312/2021v2.
• Washing effects were evaluated on 121 pesticide residues in rice.
• A significant reduction in pesticide residues was observed after washing.
• Washing is recommended to decrease pesticide residue intake and bioaccumulation.
• Pesticides reduction was not related to water solubility or chemical structure.

## 1 Introduction

Insects, weeds, and other pests that limit plant development are prevented, controlled, and removed with the use of pesticides, which can be synthetic or natural substances. These substances are categorized based on their chemical structure, applications, hazards and modes of action (Mohd Nizam et al., 2023).

Since rice is a staple food in most countries, it will always be the most extensively produced crop. However, it should be noted that rice alone cannot fulfil all the components required for proper nutrition (Tauseef et al., 2021a). Depending on whether the rice is grown in an upland, deep water, rainfed, or irrigated environment, four different agricultural rice methods are used. The most popular and preferred way of cultivating rice is irrigation because it offers a steady and dependable supply of water, which is essential for rice development. In addition, irrigation helps keep weeds and pests under control and supplies nutrients to the plants, ensuring that the rice fields are fed over the growing season. It is advised to use pesticides since the rice-cultivation environment is conducive to the growth of disease-causing pathogens, insects, weeds, and fungi (Mohd Nizam et al., 2023). Application modes could influence the efficiency of the pesticide, its distribution within the crop or soil, and its effects on non-target organisms. In other words, there are systemic and contact (non-systemic) pesticides. Pesticides classified as systemic are those that penetrate into the tissues of plants, including the stems, leaves, roots, and flowers. Systemic pesticides are absorbed by the plant's vascular system and dispersed internally, in contrast to contact pesticides, which remain on the outside of the plant. Systemic insecticides are absorbed by plants and deliver a safeguard against insects that feed on the plant's tissues. Examples of systemic pesticides include imidacloprid, clothianidin, etc. Non-systemic pesticides are pesticides that do not migrate or translocate within the plant once they are applied. These pesticides remain on the plant's surface and are neither absorbed or dispersed within its tissues, like carbaryl, cadusafos, and triflumuron (Ahmad et al., 2024).

To guarantee Public Safety as well as domestic and international trade, it is crucial to control pesticide residues in food. In fact, the analysis of pesticides in cereals is vital for ensuring traceability in the food manufacturing industry, as it helps maintain safety, comply with stringent regulations, and protect brand reputation by preventing potentially harmful products from reaching consumers. In the European Union, this is achieved by stringent legislation. Regulation (EC) No. 396/2005 and its amendments outline the guidelines for EU pesticide usage. Within this regulation, the European Commission has established standardized Maximum Residue Levels (MRL) to harmonize the regulations across Member States and prevent discrepancies in MRLs for the same pesticide (European Commission, 2005).

Therefore, the main objective of this work was to validate an HPLC-MS/MS method for determining different classes of pesticides, carbamates, organophosphates, organochlorines, and pyrethroids following the guidance document SANTE/11312/2021v2. The validation used QuEchERS as an extraction method, one of the most widely adopted methods for sample preparation in pesticide detection, primarily on fruits and vegetables (Cho et al., 2016; Belarbi et al., 2021). After the validation of 121 pesticides, it was found interesting to evaluate the effect of washing with water on the reduction of pesticides in long grain rice. There aren't many food processing techniques available that can lower the amount of pesticide residues in fruits and vegetables. Washing, cooking, ozone treatment, chilling, and ultrasonic cleaning are all efficient techniques. The first step in reducing pesticide residues on a commodity's surface is washing. The mechanism of action, chemical properties, water solubility of pesticides, and harvesting periods all affect how effective the washing procedure is. Therefore, is easier to remove contact pesticides than systemic pesticides. When it comes to lowering pesticide residues on fruits and vegetables, water solubility is crucial. Higher soluble pesticides may easily be removed, with the exception of some pesticides (Tiryaki, O., & Polat, B. (2023). The validated method is a low-cost analytical method accurately quantifying and confirming pertinent pesticides in rice.

## 2 Materials and methods

### 2.1 Chemicals and reagents

Methanol, acetonitrile (both HPLC gradient grade), toluene, acetone, n-hexane, ethyl acetate, and formic acid were acquired from Merck (Darmstadt, Germany). Water was purified using the Milli-Q Plus system from Millipore (Molsheim, France), achieving a resistivity of 18.2 MΩ·cm at 25 °C. Pesticide standards, along with internal standards (triphenylphosphate - TPP and dinitrocarbanilide or 1,3-bis(4-nitrophenyl)urea - DNC), were obtained from Sigma-Aldrich (Madrid, Spain). Based on each compound's solubility, they were prepared at a concentration of 1 μg/mL in toluene, acetone, ethanol, ethyl acetate, methanol, n-hexane, or acetonitrile. After preparing the stock solutions, working solutions were prepared in acetonitrile, a mix solution of pesticides with a concentration of 5 ng/μL, 2.5 ng/μL and 1 ng/μL and, a mix solution of internal standards with a concentration of 5 ng/μL, 2.5 ng/μL and 1 ng/μL. For QuEChERS, magnesium sulfate and sodium chloride were acquired from Fluka (Seelze, Germany). Sodium citrate dibasic sesquihydrate was purchased from Sigma–Aldrich (Madrid, Spain). Tri‑sodium citrate dihydrate was obtained from AppliChem (Darmstadt, Germany). Primary secondary amine (PSA) bonded silica, sourced from Supelco (Supelclean™, Bellefonte, PA, USA), was utilized for the clean-up process. Anhydrous magnesium sulfate was also purchased from AppliChem (Darmstadt, Germany). Mineral water was employed for the contamination of rice and for washing purposes.

### 2.2 Samples and sampling procedure

Five samples of rice were purchased in different supermarkets in Portugal between April and June of 2023 to determine pesticide residues. Rice belongs to the types: long-grain rice. Each laboratory sample (1 kg) was homogenized by grinding (Retsch rotor mill SK 300 with a sieve of trapezoid holes of 1.00 mm), and the flours were mixed carefully to ensure complete homogenization. Each sample was placed in separate sample collection tubes (50 g approx.) and preserved at −20 °C until analysis. The samples did not contain pesticide residues (blank samples), and therefore used for method validation and for artificial contamination for the washing study.

### 2.3 Methodology

#### 2.3.1 Extraction

A QuEChERS procedure was employed to extract pesticides from rice. Initially, a 10 g sample was weighed into a 50 mL polypropylene tube. Subsequently, the sample was hydrated with 20 mL of cold water and allowed to stand for 1 h. Then, 10 mL of acetonitrile (ACN) was introduced. The sample and the extractant were mixed in a vortex for 1 min.

Next, a mixture of extraction salts for the liquid-liquid partitioning step was added, comprising 4 g of magnesium sulfate (MgSO4), 1 g of sodium chloride (NaCl), 1 g of sodium citrate, and 0.5 g of disodium hydrogen citrate sesquihydrate, followed by an additional 1 min of mixing in a vortex. The mixture was then centrifuged at 12,669 ×*g* for 5 min at 5 °C. After centrifugation, 6 mL of the supernatant was transferred to a mixture with primary secondary amine (PSA) and 1.05 g of anhydrous magnesium sulfate, a step known as dispersive solid-phase extraction (dSPE) for clean-up.

After thorough mixing and a subsequent centrifugation at 12,669 ×*g* for 2 min, 1 mL of the extract was combined with 220 μL of ACN in an Eppendorf tube. Finally, 25 μL of a mixture of internal standards (TPP and DPP) was added to 500 μL of the extract in a mini-uniprep™. The resulting extract was then analyzed using high-performance liquid chromatography-tandem mass spectrometry (HPLC-MS/MS) with a triple quadrupole mass spectrometer utilizing electrospray ionization (ESI).

#### 2.3.2 Matrix-matched calibration

A matrix-matched calibration curve was prepared with 10 concentration levels, i.e., at 5, 10, 20, 30, 40, 50, 60, 70, 80, and 100 μg/kg. Before this, a solvent calibration curve is prepared with defined volumes of the mix of internal standards at 2.5 ng/μL, a working solution at 250 μg/kg (the pesticides that are in this solution are found in Table 1) in ACN (5–200 μg/kg). Then, 500 μL is taken from the levels of the solvent calibration curve and 500 μL of a blank sample and 10 μL of a solution of internal standards at 2.5 ng/μL are added to each level (5 to 100 μg/kg). For instance, for the construction of the 5 μg/kg level, 500 μL is taken from the 10 μg/kg level of the solvent curve and added to 500 μL of a blank sample and 10 μL of a solution of internal standards. Following, each vial is injected into the HPLC-MS/MS.

**Table 1:** Results of the validation of the HPLC-MS/MS method to determine 121 pesticides in rice: determination coefficient (r^2^) in matrix-matched curves, recovery, repeatability (RSD_r_) and precision (RSD_R_), limit of quantification (LOQ) and expanded uncertainty (U).

Pesticide	Linear Range (μg/L)	R^2^ matrix	LOQ (μg/kg)	RSD_R_	RSD_r_	Recovery (%)	U (%)
Acetamiprid	10–100	0.9917	10	8.34	7.36	113.5	12
Azoxystrobin	10–100	0.9834	10	13.9	7.02	91.82	39
Bitertanol	10–100	0.9848	10	13.1	13.6	102.8	19
Bixafen	10–100	0.9830	10	11.0	6.16	80.40	30
Boscalid	5–100	0.9874	5	11.4	7.50	81.25	29
Bupirimate	10–100	0.9786	10	8.48	6.79	98.89	12
Buprofezin	10–100	0.9774	10	15.5	6.98	90.69	49
Cadusafos	5–100	0.9885	5	12.9	7.01	94.50	36
Carbaryl	10–100	0.9868	10	10.6	6.21	103.5	26
Carbendazim	10–100	0.9747	10	16.3	14.6	70.02	32
Carbofuran	10–100	0.9830	10	8.03	6.38	107.9	13
Carbofuran-3-hydroxy	10–100	0.9830	10	17.0	10.9	103.7	43
Carboxin	10–100	0.9841	10	7.7	6.37	103.2	10
Chlorantraniliprole	10–100	0.9819	10	8.1	6.81	109.1	13
Chlorfenvinphos	10–100	0.981	10	12.3	6.34	82.10	35
Chlorpyrifos-methyl	10–100	0.9958	10	15.2	7.07	92.22	38
Clofentezine	10–100	0.9831	10	9.13	5.90	83.99	20
Coumaphos	50–100	0.9720	50	14.4	8.86	80.90	31
Cymoxanil	10–100	0.9983	10	13.4	12.31	104.1	22
Cyproconazole	10–100	0.9782	10	8.23	6.17	117.1	14
Cyprodinil	10–100	0.9809	10	9.84	5.89	89.96	25
Demeton-*S*-methylsulfone	10–100	0.9800	10	15.9	11.3	103.8	39
Diazinon	10–100	0.9854	10	8.93	5.23	101.4	23
Dichlorvos	10–100	0.9825	10	11.3	7.41	105.7	31
Difenoconazole	10–100	0.9756	10	13.5	7.48	100.7	39
Diflubenzuron	10–100	0.9814	10	11.6	6.67	85.69	29
Dimethoate	5–100	0.9874	5	12.4	9.72	102.9	32
Dimethomorph	10–100	0.9768	10	12.1	6.96	99.78	26
Diniconazole	10–100	0.9810	10	12.6	7.49	106.8	38
DMST	10–100	0.9842	10	10.2	6.51	101.1	28
EPN	10–100	0.9766	10	8.49	6.99	89.67	9
Epoxiconazole	10–100	0.9759	10	10.4	7.37	110.0	19
Ethiofencarb	10–100	0.9864	10	8.54	6.48	110.4	17
Ethoprophos	5–100	0.9857	5	12.9	6.26	91.34	37
Etrimfos	50–100	0.9767	50	8.08	6.97	90.23	15
Fenamidone	10–100	0.9774	10	10.4	7.06	109.8	25
Fenamiphos sulfone	5–100	0.9880	5	7.02	6.31	94.75	12
Fenamiphos sulfoxide	10–100	0.9809	10	7.12	5.50	100.4	8
Fenarimol	10–100	0.9750	10	10.2	7.66	96.38	23
Fenitrothion	10–100	0.9759	10	14.6	9.55	96.56	32
Fenoxycarb	10–100	0.9777	10	13.6	6.17	109.5	39
Fenpropathrin	10–100	0.9750	10	9.19	3.56	78.34	20
Fenpropidin	5–100	0.9839	5	9.42	7.68	100.9	13
Fenpropimorph	10–100	0.9778	10	8.72	7.56	105.0	8
Fenpyroximate	50–100	0.9639	50	13.6	9.79	91.31	24
Fenthion oxon sulfone	10–100	0.9839	10	10.2	8.01	114.2	19
Fenthion oxon sulfoxide	10–100	0.9845	10	9.18	7.30	108.7	15
Fenthion oxon	10–100	0.9812	10	13.5	8.18	99.60	35
Fenthion sulfone	10–100	0.9863	10	9.14	5.74	104.3	21
Fenthion sulfoxide	10–100	0.9844	10	10.9	6.87	109.1	28
Fluopyram	5–100	0.9863	5	8.80	6.06	99.78	21
Fluquinconazole	10–100	0.9788	10	13.7	7.18	87.06	33
Flutriafol	5–100	0.9864	5	15.7	7.99	106.4	46
Fonofos	50–100	0.9798	50	9.71	14.5	80.60	31
Fosthiazate	10–100	0.9801	10	9.58	6.62	111.2	20
Hexythiazox	10–100	0.9743	10	14.0	9.18	90.38	28
Indoxacarb	10–100	0.9761	10	8.92	6.19	109.8	19
Iprodione	10–100	0.9781	10	11.8	6.72	72.66	29
Iprovalicarb	10–100	0.9765	10	8.66	6.31	107.2	15
Isoprocarb	10–100	0.9791	10	9.67	6.10	103.9	23
Isoprothiolane	50–100	0.9802	50	11.1	7.33	101.9	24
Isoproturon	10–100	0.9817	10	10.6	5.75	98.80	28
Kresoxim-methyl	10–100	0.9772	10	13.8	8.66	118.1	36
Linuron	10–100	0.9845	10	7.65	5.85	100.4	13
Lufenuron	5–100	0.9817	5	15.3	9.83	73.05	46
Malaoxon	10–100	0.9827	10	15.3	7.85	97.47	48
Malathion	10–100	0.9760	10	10.5	6.95	91.70	24
Mepanipyrim	10–100	0.9823	10	10.9	6.45	85.49	28
Metaflumizone	50–100	0.9790	50	10.8	6.90	79.90	20
Metalaxyl	5–100	0.9804	5	6.62	5.39	104.4	8
Metalaxyl-M	10–100	0.9810	10	8.29	6.01	100.6	12
Metazachlor	10–100	0.9818	10	12.3	6.64	109.7	34
Metconazole	10–100	0.9815	10	11.5	7.69	113.9	27
Methiocarb	10–100	0.9828	10	14.4	6.96	90.97	44
Methomyl	10–100	0.9772	10	13.7	11.5	100.3	32
Metribuzin	10–100	0.9897	10	9.79	7.96	105.4	18
Mevinphos	5–100	0.9878	5	13.9	9.43	108.7	34
Monocrotophos	5–100	0.9842	5	12.5	9.83	97.15	32
Oxadixyl	10–100	0.9673	10	9.08	7.00	108.6	17
Paclobutrazole	10–100	0.9821	10	10.3	6.76	115.7	25
Paraoxon-ethyl	10–100	0.9845	10	10.2	6.51	94.53	22
Paraoxon-methyl	10–100	0.9803	10	9.80	7.41	100.7	17
Parathion	10–100	0.9779	10	12.9	7.18	97.65	37
Parathion-methyl	10–100	0.9811	10	14.9	8.19	89.33	40
Penconazole	10–100	0.9819	10	7.31	11.3	110.0	30
Pencycuron	10–100	0.9811	10	16.5	8.55	102.7	47
Pendimethalin	50–100	0.9760	50	13.2	13.70	73.72	28
Phenthoate	10–100	0.9802	10	9.20	7.06	87.69	14
Phosalone	10–100	0.9827	10	10.9	8.24	76.55	27
Phosmet	10–100	0.9788	10	12.5	5.31	97.77	36
Phosphamidon	10–100	0.9837	10	8.00	6.37	100.4	12
Phoxim	5–100	0.9831	5	12.3	8.07	100.6	35
Pirimiphos-ethyl	50–100	0.9532	50	11.5	8.36	83.72	21
Pirimiphos-methyl	5–100	0.9868	5	10.8	6.88	93.54	29
Prochloraz	10–100	0.9781	10	12.7	5.80	91.00	37
Profenofos	10–100	0.9747	10	17.1	7.46	94.80	49
Propiconazole	10–100	0.9799	10	9.01	6.82	96.89	13
Propoxur	10–100	0.9830	10	9.95	7.33	103.9	23
Propyzamide	10–100	0.9811	10	9.94	5.62	96.97	24
Prothioconazole-desthio	10–100	0.9803	10	9.35	8.02	111.3	15
Pyrazophos	5–100	0.9869	5	12.8	9.56	80.78	24
Pyrimethanil	10–100	0.9809	10	7.92	6.84	96.80	10
Quinoxyfen	5–100	0.9872	5	13.4	9.59	92.62	31
Rotenone	10–100	0.9815	10	11.7	7.37	86.62	29
SpinosadA	5–100	0.9866	5	14.6	7.33	97.08	46
SpinosadD	10–100	0.9830	10	8.55	5.24	108.8	20
Spiroxamine	5–100	0.9862	5	12.0	7.50	95.58	25
Tebuconazole	50–100	0.9797	50	8.96	6.43	98.45	16
Tebufenpyrad	10–100	0.9848	10	13.3	9.44	78.46	34
Terbuthylazine	10–100	0.9809	10	10.1	6.64	91.52	24
Tetraconazole	10–100	0.9856	10	12.9	7.96	93.76	32
Thiabendazole	10–100	0.9761	10	19.7	16.0	112.7	49
Thiacloprid	10–100	0.9882	10	9.18	6.53	113.2	20
Thiodicarb	10–100	0.9819	10	11.1	8.79	83.66	16
Triadimefon	10–100	0.9805	10	10.6	7.92	116.4	22
Triadimenol	10–100	0.9768	10	10.3	7.87	119.1	19
Tricyclazole	10–100	0.9800	10	6.62	5.93	96.80	10
Triflumuron	10–100	0.9786	10	9.48	5.24	74.62	19
Zoxamide	5–100	0.9881	5	8.69	5.77	80.53	20
Fludioxonil	10–100	0.9740	10	11.9	6.10	80.95	36

**Legend:** DMST- *N*,*N*-dimethyl-N′-p-tolysulphamide; EPN- O-ethyl O-4-nitrophenyl phosphonothiate; U- expanded uncertainty.

#### 2.3.3 Spiking experiment

Spiking experiments were performed to determine the recovery of the target analytes. Blank samples of rice (10 g) were spiked at three different concentrations (5,10 and 50 μg/kg) using a multi-pesticide standard solution in acetonitrile (*v*/v). After spiking, the solution was left in contact with the matrix at room temperature in the dark for 30 min. Subsequently, extraction was performed as described in Section 2.3.1.

#### 2.3.4 Sample preparation for processing

For this step, the paper of Shakoori et al. (2018) was used as guidance and adapted. Before the cooking process, two solutions of pesticides, one at a concentration level of 20 and another at 50 μg/L, were prepared for contamination of the long grain rice at two levels in a beaker with water. For the contamination level of 20 μg/kg, 1.6 mL of mixed pesticide solution (5000 μg/kg) was dissolved in 400 mL of water. For the contamination level of 50 μg/kg, 4 mL of mixed pesticide solution (5000 μg/kg) was dissolved in 400 mL of water. Then, 200 g of long grain rice was submerged, followed by air-drying at room temperature for 24 h. To dry the rice, it was spread out on trays and left in the sun until completely dry. For the washing process, 20 g of rice was used.

##### 2.3.4.1 Washing

A 20 g portion of the rice samples was washed with mineral water and soaked in 100 mL for 20 min. The samples were grounded and then analyzed.

### 2.4 HPLC-MS/MS parameters

A UHPLC Nexera X2 system (Shimadzu, Kyoto, Japan) coupled with a QTRAP 5500+ MS/MS detector (AB SCIEX, Foster City, CA, USA) was utilized for detection and quantification. This setup featured an electrospray ionization source operating in both positive and negative modes (ESI+ and ESI-). The autosampler was maintained at 10 °C to keep the samples chilled, and a 10 μL volume of the sample extract was injected into the analytical column under the following chromatographic conditions: a Synergi 4 μm Fusion-RP 80 A 50 × 2 mm column (Phenomenex, Torrance, CA, USA) was employed.

The mobile phase was a gradient composed of formic acid 0.1 % in ultrapure water as mobile phase [A] and formic acid 0.1 % in methanol as mobile phase [B], at a flow rate of 0.25 mL/min. The gradient elution program starts with 5 % [B] for 0.5 min and then up to 90 % [B] until 8 min and keep at 90 % [B] for five minutes. Then, decreased for initial conditions in 2 min and keep at 5 % [B] for 3 min. The total analysis time was 18 min. Mass spectrometry data were collected in multiple reaction monitoring (MRM) mode over a range of 100 to 750 Da using Analyst® TF software (SCIEX, Foster City, CA, USA) with the following settings: ion spray voltage at 4500 V, source temperature at 600 °C, curtain gas (CUR) at 35 psi, and gas 1 and gas 2 set at 40 and 60 psi, respectively. Parameters for determining pesticide residues in rice using MS/MS in ESI+ and ESI- modes are detailed in Table A1, Table A2. Data acquisition was calibrated through direct injection of each standard solution at a concentration of 1 μg/mL into the detector, with two ion transitions selected for each compound: one for quantification and one for qualification in MRM mode.

### 2.5 Identification of pesticide residues in rice

The identification and data processing of pesticide residues in rice were made through the MultiQuant™ software (SCIEX, Foster City, CA, USA).

In accordance with SANTE/11312/2021v2, two parameters were applied as identification criteria: the ion ratio tolerance, which was set to be below 30 %, and the retention time (RT) tolerance, which was established at 0.1 min relative to the RT of the analyte in the calibration standard (potentially requiring matrix matching). For this analysis, two internal standards were employed: TPP for residues of pesticides detected in positive mode and DNC for those identified in negative mode.

Eq. (1): Deviation of RRT,

(1) $$\Delta \mathrm{RRT}=\left({\mathrm{RT}}_{\mathrm{sample}}-{\mathrm{RT}}_{\mathrm{mean calibration}}\right),$$

where RT_sample_ is the retention time of the analyte in a sample, and RT_mean calibration_ corresponds to the mean of retention time obtained for the same analyte in a set of calibrations (matrix-matched calibration curves were used). The ratio between the areas measured for both ion transitions of each analyte is used to calculate the ion ratio.

Eq. (2): Ion ratio (IR, %),

(2) $$\mathrm{IR}=\left(\frac{A_{\mathrm{ion}\;\text{with lowest intensity}}}{A_{\mathrm{ion}\;\mathrm{with highest intensity}}}\right)\times 100$$

where, A_ion with lowest intensity_ corresponds to the area of the ion with the lowest intensity and the A_ion with highest intensity_ to the area of the ion with the highest intensity.

Eq. (3): Deviation of IR (∆IR, %),

(3) $$∆\mathrm{IR}=\frac{{\mathrm{IR}}_{\mathrm{Sample}}-{\mathrm{IR}}_{\mathrm{mean calibrat}i\mathrm{on}}}{{\mathrm{IR}}_{\mathrm{mean calibration}}}\times 100,$$

where IR_mean calibration_ refers to the mean ion ration achieved for a batch of calibration of the same analyte, and IR_sample_ corresponds to the ion ratio obtained for a target compound present in a sample.

The positive identification is reached if both criteria are fulfilled (∆RRT < 0.1 min and ∆IR < 30 % - Eqs. (2), (3)).

### 2.6 Validation of HPLC–MS/MS method

The method was validated based on various parameters, including specificity, working range, linearity, limit of quantification (LOQ), precision (both repeatability and intra-laboratory reproducibility), and accuracy (assessed via recovery assays). Additionally, expanded uncertainty was evaluated.

To determine repeatability (RSDr) and intra-laboratory reproducibility (RSDR), blank rice samples were spiked at three different concentration levels (*n* = 5), taking into consideration the maximum residue limits (MRL) for each pesticide. For assessing RSDR, experiments were conducted over three different days by different operators. The accuracy of the method was verified through recovery assays and the use of certified reference materials.

### 2.7 Processing factors (PF) evaluation

Pesticide residue reduction during processing, i.e. washing, was evaluated by calculating the processing factor (PF) according to the equation,

PFs = $\frac{C_{a}}{C_{b.}}$

Where,

Ca = concentration of pesticide in washed samples (mg/kg)

C_b_ = concentration of pesticide in raw samples (mg/kg)

PF < 1, it means the reduction of pesticide concentration

PF >1, it means the increase of pesticide residue concentration

The percentage reduction (% Re) was calculated according to the equation,

%Re=1−PFs×100

### 2.8 Statistical analysis

The statistical analysis of the data was analyzed with IBM® SPSS® Statistics, version 28.0.1.1. (Chicago, IL, USA). For the evaluation of washing effect, *t*-Test was applied to compare the concentration of each pesticide before and after washing, when the normality and homogeneity of variances were validated. Significance was defined at *p* < 0.05. Results concerning the statistical evaluation were expressed as mean value plus the standard deviation (SD) of three replicates (*n* = 3).

## 3 Results and discussion

### 3.1 Validation of the method

The method was validated according to the criteria defined by SANTE/11312/2021v2, which establishes the validation parameters for the official control of the pesticides in foodstuffs in the EU, and 121 pesticides in total were validated (SANTE, 2021).

Linearity was evaluated by matrix-matched calibration curves (mean of six curves) in different ranges for different pesticide residues (see Table 1). The linear range of the calibration curves ranged between 5 and 100, 10–100 or 50–100 μg/L, depending on the pesticide. The limit of quantification was 5, 10, or 50 μg/kg, which is sensitive enough to meet the requirements imposed by EU regulations for the MRL of pesticide residues in rice (European Commission, 2005). The determination coefficients varied between 0.9639 and 0.9983, indicating suitability for pesticide quantification, except for pirimiphos-ethyl, which presented 0.9532. Table 1 shows the linearity, repeatability, precision, recovery, and expanded uncertainty results for the different pesticide residues determined at the LOQ level. Recoveries determined after spiked blank samples at three concentration levels ranged between 70.0 and 119 % for the 121 analyzed pesticides. The specificity criteria were met for all pesticides at 5 μg/kg, except for chlorantraniliprole, chlorfenvinphos and metazachlor, where they were met at 10 μg/kg, and for hexythiazox and fludioxanil, where they were met at 50 μg/kg.

The recoveries of the method were all within the appropriate range of the SANTE/11312/2021v2 criteria. The repeatability of the method was evaluated by the Relative Standard Deviation RSD_r_. RSD_r_ was between 5.71 and 17.1 %. The reproducibility was assessed by the Relative Standard Deviation RSD_R_ on 3 different days of analysis; different concentration levels and values were considered acceptable (varied between 6.62 and 19.7 %).

There is a vast literature on the extraction of pesticides from rice (de Arias et al., 2014; Teló et al., 2017); however, there are many different results from each other. Tauseef et al. (2021a) validated a method that allows to determine 25 pesticides residues in rice, using a slightly different QuEChERS extraction method (acetate buffered QuEChERS method without PSA cleanup), and obtained excellent results, for example, for the coefficient of determination of the matrix curve, they had results between 0.994 and 0.999, also using LC-MS/MS. The selected 25 pesticides residues met the EU-SANTE and FAO/WHO Codex Alimentarius Commission method validation guidelines. The interday repeatability of the optimized method was between 4 and 18 % (*n* = 6). The expanded uncertainty calculated for the optimized method ranged from 22 to 48 % (Tauseef et al., 2021b).

Whereas Tsochatzis et al. (2010) used MSPD extraction for the determination of nine pesticides residues instead of the traditional QuEChERS, also allowing to obtain good results for the recovery (97.1–104.6 %) and linearity (coefficient of determination: 0.9948–0.9999). Samples were further analyzed by high-performance liquid chromatography coupled with diode array detection (HPLC-DAD) (Tsochatzis et al., 2010).

An excellent study by Tran-Lam et al. (2021) determined 656 pesticide residues using UHPLC-MS/MS and GC–MS/MS combined with QuEChERS extraction and mixed-mode SPE clean-up. Validating the method followed SANTE/12682/2019. Linear regressions of all analyzes exhibit coefficient of determination greater than 0.999, which indicates excellent goodness-of-fit for the calibration points. All analyzes displayed recovery between 70 and 120 % with RSD_r_ and RSD_R_ less than 20 %. Furthermore, the maximal LOQs were 10 μg/kg in both MS methods (SANTE, 2019; Tran-Lam et al., 2021).

At our laboratory, a method had already been validated following SANTE/11813/2017 for validating pesticide residues quantification in rice (Melo et al., 2020). This method also allowed to achieve good results for all parameters; for example, recoveries varied between 77.1 and 111.5 % and with a linear range between 5 and 50, 5–60, or from 5 to 70 μg/kg, depending on the pesticide (Melo et al., 2020). The present method allows the quantification of pesticide residues in rice at wide range of concentration levels (up to 100 μg/kg). The QuEChERS method that has been used in this paper differs from the one original QuEChERS method due to include an hydration step with cold water, improving extraction efficiency for certain pesticides and due to use a specific salt mixture for liquid–liquid partitioning, which enhances extraction performance and selectivity. This is composed of 4 g MgSO4, 1 g NaCl, 1 g sodium citrate, and 0.5 g disodium hydrogen citrate sesquihydrate) while the original one used MgSO4 and NaCl. Moreover, the centrifugation is carried out at low temperature for better phase separation.

Nowadays, it is increasingly important to develop analytical methodologies for detecting multi-pesticide residues at very low limits of quantification to evaluate if food samples meet the established maximum residue levels at the EU level.

### 3.2 Effect of washing process on pesticide residues in rice

The study of pesticide residues in various commodities, including rice, is a major challenge in food safety. Rice is processed by different methods all around the world. So, it is important to evaluate the effect of different processes, such as washing and cooking, on the levels of pesticide residues.

The study aimed to analyze the effect of washing in reducing pesticides in long-grain rice samples.

#### 3.2.1 Unprocessed samples

It is important to ascertain the pesticide concentration in unprocessed rice samples to calculate the pesticide reduction throughout the washing processes. Three replicates of each rice sample (unprocessed and washed) are shown in Table 2, Table 3, for each contamination level, respectively.

**Table 2:** Mean concentrations (± SD, *n* = 3), mean values of processing factors (PFs) and reductions (%) of the pesticides in unprocessed rice samples (20 μg/kg), after washing.

Pesticides	Unprocessed Samples	Washing		
	Concentration ± SD	Concentration ± SD	PF	Reduction (%)
Acetamiprid	<LOQ	<LOQ	–	–
Azoxystrobin	6.49 ± 0.39	<LOQ	–	> 23.0
Bixafen	12.4 ± 1.37	10.5 ± 1.25	0.84	15.6
Boscalid	7.42 ± 0.84	<LOQ	–	> 32.6
Bupirimate	<LOQ	<LOQ	–	–
Buprofezin	10.8 ± 0.67	<LOQ	–	> 7.24
Cadusafos	7.15 ± 0.82	<LOQ	–	> 30.1
Carbaryl	<LOQ	<LOQ	–	–
Carbendazim	<LOQ	<LOQ	–	–
Carbofuran	<LOQ	<LOQ	–	–
Carboxin	<LOQ	<LOQ	–	–
Chlorantraniliprole	<LOQ	<LOQ	–	–
Chlorfenvinphos	13.0 ± 2.30	12.1 ± 1.12	0.93	6.9
Chlorpyrifos-methyl	<LOQ	<LOQ	–	–
Coumaphos	<LOQ	<LOQ	–	–
Cyprodinil	10.4 ± 1.57	<LOQ	–	> 3.85
Diazinon	16.1 ± 0.21	15. 2 ± 0.31 *	0.94	5.8
Difenoconazole	<LOQ	<LOQ	–	–
Diflubenzuron	13.0 ± 0.84	10.9 ± 0.77	0.84	16.2
Dimethoate	5.39 ± 0.08	5.38 ± 0.34	0.99	1
Dimethomorph	<LOQ	<LOQ	–	–
Diniconazole	13.5 ± 1.81	12.5 ± 0.32	0.93	6.8
DMST	<LOQ	<LOQ	–	–
EPN	<LOQ	<LOQ	–	–
Epoxiconazole	12.2 ± 1.61	9.86 ± 0.38 *	0.81	19.1
Ethoprophos	<LOQ	<LOQ	–	–
Etrimfos	<LOQ	<LOQ	–	–
Fenamidone	<LOQ	<LOQ	–	–
Fenamiphos sulfone	<LOQ	<LOQ	–	–
Fenamiphos sulfoxide	<LOQ	<LOQ	–	–
Fenarimol	12.4 ± 2.43	11.1 ± 0.55	0.89	10.8
Fenoxycarb	<LOQ	<LOQ	–	–
Fenpropidin	11.5 ± 1.98	<LOQ	–	> 12.7
Fenpropimorph	14.5 ± 2.09	10.1 ± 0.05 *	0.70	30.4
Fenthion oxon sulfone	<LOQ	<LOQ	–	–
Fenthion oxon sulfoxide	<LOQ	<LOQ	–	–
Fenthion oxon	<LOQ	<LOQ	–	–
Fenthion sulfoxide	12.1 ± 1.22	<LOQ	–	> 17.0
Fluopyram	<LOQ	<LOQ	–	–
Fluquinconazole	11.8 ± 1.22	10.7 ± 0.37	0.91	9.5
Flutriafol	8.30 ± 1.53	6.27 ± 0.33 *	0.76	24.4
Fonofos	<LOQ	<LOQ	–	–
Fosthiazate	<LOQ	<LOQ	–	–
Hexythiazox	<LOQ	<LOQ	–	–
Indoxacarb	11.6 ± 0.48	<LOQ	–	> 14.1
Iprodione	<LOQ	<LOQ	–	–
Iprovalicarb	<LOQ	<LOQ	–	–
Isoprocarb	<LOQ	<LOQ	–	–
Isoprothiolane	<LOQ	<LOQ	–	–
Isoproturon	<LOQ	<LOQ	–	–
Linuron	10.5 ± 1.39	<LOQ	–	> 5.12
Lufenuron	<LOQ	<LOQ	–	–
Mepanipyrim	16.7 ± 1.47	13.5 ± 0.87 *	0.81	18.7
Metaflumizone	<LOQ	<LOQ	–	–
Metalaxyl	<LOQ	<LOQ	–	–
Metalaxyl-M	<LOQ	<LOQ	–	–
Metazachlor	<LOQ	<LOQ	–	–
Metconazole	14.1 ± 0.80	13.0 ± 0.68	0.92	8.0
Methiocarb	10.6 ± 2.19	10.2 ± 0.53	0.96	4.14
Metrobromuron	<LOQ	<LOQ	–	–
Metribuzin	<LOQ	<LOQ	–	–
Oxadixyl	<LOQ	<LOQ	–	–
Paclobutrazole	11.2 ± 1.81	<LOQ	–	> 10.55
Parathion	<LOQ	<LOQ	–	–
Parathion-methyl	<LOQ	<LOQ	–	–
Penconazole	14.7 ± 1.52	13.1 ± 1.20	0.89	11.22
Pencycuron	<LOQ	<LOQ	–	–
Phenthoate	13.5 ± 1.07	<LOQ	–	> 25.7
Phosphamidon	<LOQ	<LOQ	–	–
Pirimiphos-ethyl	<LOQ	<LOQ	–	–
Pirimiphos-methyl	<LOQ	<LOQ	–	–
Prochloraz	<LOQ	<LOQ	–	–
Profenofos	<LOQ	<LOQ	–	–
Propiconazole	11.6 ± 3.29	<LOQ	–	> 13.7
Propyzamide	11.2 ± 0.99	10.5 ± 0.74	0.94	6.22
Prothioconazole-desthio	11.0 ± 2.09	<LOQ	–	> 8.84
Pyrazophos	13.2 ± 1.10	12.8 ± 0.04	0.97	2.96
Pyrimethanil	12.6 ± 1.23	11.4 ± 0.62	0.90	9.66
Quinoxyfen	<LOQ	<LOQ	–	–
Rotenone	<LOQ	<LOQ	–	–
SpinosadA	9.73 ± 1.51	7.43 ± 0.22 *	0.76	23.7
SpinosadD	<LOQ	<LOQ	–	–
Spiroxamine	12.1 ± 1.82	8.79 ± 0.54 *	0.73	23.7
Tebuconazole	<LOQ	<LOQ	–	–
Tebufenpyrad	12.8 ± 1.48	11.0 ± 0.88	0.86	13.5
Terbuthylazine	12.5 ± 1.93	<LOQ	–	> 19.7
Tetraconazole	11.9 ± 3.10	<LOQ	–	> 16.0
Thiabendazole	<LOQ	<LOQ	–	–
Thiacloprid	<LOQ	<LOQ	–	–
Triadimefon	13.5 ± 0.57	<LOQ	–	> 25.9
Triadimenol	12.4 ± 1.40	<LOQ	–	> 19.2
Tricyclazole	<LOQ	<LOQ	–	–
Triflumuron	<LOQ	<LOQ	–	–
Zoxamide	12.6 ± 0.47	12.6 ± 0.84	0.99	0.53
Fludioxonil	11.5 ± 1.09	<LOQ	–	> 13.0

**Legend:** DMST- *N*,*N*-dimethyl-N′-p-tolysulphamide; EPN- O-ethyl O-4-nitrophenyl phosphonothiate.

The asterisk (*) means a significant difference (*p* < 0.05) between the concentration of unprocessed and washed samples, using the Student's t-test.

**Table 3:** Mean concentrations (±SD, n = 3), mean values of processing factors (PFs) and reductions (%) of the pesticides in unprocessed rice samples (50 μg/kg), after washing.

Pesticides	Unprocessed Samples	Washing		
	Concentration ± SD	Concentration ± SD	PF	Reduction (%)
Acetamiprid	19.2 ± 1.93	12.5 ± 1.87*	0.65	34.7
Azoxystrobin	32.9 ± 4.50	13.2 ± 2.82*	0.40	59.8
Bixafen	32.7 ± 0.54	24.3 ± 1.71*	0.74	25.8
Boscalid	28.0 ± 2.69	15.0 ± 1.01*	0.54	46.4
Bupirimate	35.2 ± 4.89	16.1 ± 2.31*	0.46	54.2
Buprofezin	34.4 ± 2.47	19.5 ± 1.38*	0.57	43.4
Cadusafos	34.5 ± 6.30	11.8 ± 0.91*	0.34	65.8
Carbaryl	25.4 ± 0.73	19.3 ± 1.35*	0.76	24
Carbendazim	24.2 ± 0.52	18.3 ± 2.36*	0.76	24.4
Carbofuran	14.6 ± 1.63	9.85 ± 1.15*	0.68	32.4
Carboxin	<LOQ	<LOQ	–	–
Chlorantraniliprole	22.8 ± 0.13	14.1 ± 0.02*	0.62	38.2
Chlorfenvinphos	36.1 ± 1.25	22.0 ± 2.28*	0.61	34
Chlorpyrifos-methyl	24.0 ± 0.23	15.9 ± 0.65*	0.66	33.8
Coumaphos	<LOQ	<LOQ	–	–
Cyprodinil	30.0 ± 1.97	14.9 ± 0.85*	0.50	50.4
Diazinon	37.3 ± 0.34	31.7 ± 1.80*	0.85	15.1
Difenoconazole	32.7 ± 5.21	12.8 ± 1.14*	0.39	61
Diflubenzuron	37.6 ± 4.07	19.2 ± 0.92*	0.51	48.9
Dimethoate	13.3 ± 1.26	10.7 ± 1.44*	0.80	20.0
Dimethomorph	26.7 ± 2.54	14.8 ± 1.67*	0.55	44.5
Diniconazole	40.6 ± 3.53	26.5 ± 1.42*	0.65	34.6
DMST	24.9 ± 0.54	13.9 ± 0.17*	0.56	44.2
EPN	21.5 ± 5.47	<LOQ	–	>53.6
Epoxiconazole	41.2 ± 4.64	21.3 ± 1.44*	0.52	48.3
Ethoprophos	27.3 ± 3.13	13.4 ± 1.60*	0.49	50.9
Etrimfos	<LOQ	<LOQ	–	–
Fenamidone	36.3 ± 5.15	12.4 ± 1.22*	0.34	65.9
Fenamiphos sulfone	14.8 ± 0.59	11.3 ± 1.61*	0.76	23.5
Fenamiphos sulfoxide	16.8 ± 1.69	<LOQ	–	>40.4
Fenarimol	39.6 ± 2.82	23.3 ± 1.51*	0.59	41.4
Fenoxycarb	35.7 ± 5.81	12.9 ± 1.66*	0.36	63.8
Fenpropidin	37.2 ± 4.15	18.4 ± 1.92*	0.49	50.7
Fenpropimorph	39.9 ± 2.73	19.1 ± 0.89*	0.48	52
Fenthion oxon sulfone	23.3 ± 3.60	9.88 ± 0.50*	0.42	57.1
Fenthion oxon sulfoxide	11.3 ± 1.15	<LOQ	–	>11.3
Fenthion oxon	23.8 ± 1.07	<LOQ	–	>58
Fenthion sulfoxide	23.2 ± 1.82	10.5 ± 0.06*	0.45	54.8
Fluopyram	32.0 ± 2.28	13.1 ± 1.24*	0.41	59.2
Fluquinconazole	31.6 ± 0.03	19.4 ± 2.59*	0.61	38.8
Flutriafol	30.9 ± 3.60	13.2 ± 0.80*	0.43	57.3
Fonofos	<LOQ	<LOQ	–	–
Fosthiazate	16.9 ± 2.22	13.8 ± 0.65*	0.81	18.7
Hexythiazox	13.2 ± 3.31	<LOQ	–	>24.4
Indoxacarb	25.3 ± 2.28	23.4 ± 4.73	0.92	7.7
Iprodione	26.9 ± 1.95	21.8 ± 2.31*	0.81	18.7
Iprovalicarb	34.7 ± 5.22	15.5 ± 1.25*	0.44	55.4
Isoprocarb	22.6 ± 1.45	15.7 ± 3.30*	0.69	30.6
Isoprothiolane	<LOQ	<LOQ	–	–
Isoproturon	22.8 ± 2.63	10.9 ± 0.73*	0.48	52.0
Linuron	28.8 ± 2.17	21.1 ± 2.08*	0.73	26.7
Lufenuron	13.6 ± 3.86	7.57 ± 1.97*	0.55	44.3
Mepanipyrim	38.4 ± 3.16	24.4 ± 2.02*	0.63	36.6
Metaflumizone	<LOQ	<LOQ	–	–
Metalaxyl	13.9 ± 2.26	9.66 ± 0.51*	0.70	30.4
Metalaxyl-M	14.8 ± 2.36	9.20 ± 0.37*	0.62	37.9
Metazachlor	21.3 ± 0.79	15.9 ± 2.09*	0.75	25.3
Metconazole	37.8 ± 1.66	24.7 ± 2.31*	0.65	34.7
Methiocarb	30.7 ± 2.36	13.0 ± 2.49*	0.55	44.7
Metrobromuron	28.1 ± 3.95	16.0 ± 0.02*	0.57	42.9
Metribuzin	20.3 ± 1.00	10.4 ± 0.69*	0.51	49.0
Oxadixyl	12.8 ± 1.03	9.25 ± 2.52	0.72	21.8
Paclobutrazole	38.6 ± 3.59	17.4 ± 1.25*	0.45	54.9
Parathion	23.0 ± 2.04	18.0 ± 1.56*	0.78	21.8
Parathion-methyl	16.3 ± 2.10	11.3 ± 0.59*	0.69	30.5
Penconazole	40.6 ± 1.35	23.8 ± 2.14*	0.59	41.4
Pencycuron	29.1 ± 3.95	17.1 ± 1.22*	0.59	41.1
Phenthoate	34.8 ± 4.92	21.6 ± 3.00*	0.62	37.9
Phosphamidon	<LOQ	<LOQ	–	–
Pirimiphos-ethyl	<LOQ	<LOQ	–	–
Pirimiphos-methyl	32.9 ± 5.57	8.86 ± 1.27*	0.27	73.0
Prochloraz	22.0 ± 6.35	11.4 ± 1.16*	0.52	48.1
Profenofos	27.4 ± 2.58	13.6 ± 1.63*	0.49	50.5
Propiconazole	41.7 ± 5.79	14.5 ± 1.72*	0.35	65.4
Propyzamide	33.3 ± 0.03	20.3 ± 3.53*	0.61	39.1
Prothioconazole-desthio	39.8 ± 3.30	15.3 ± 0.70*	0.38	61.5
Pyrazophos	34.0 ± 1.85	23.4 ± 1.31*	0.69	31.3
Pyrimethanil	36.2 ± 2.45	23.6 ± 1.52*	0.65	34.8
Quinoxyfen	12.9 ± 0.87	9.28 ± 0.42*	0.71	28.3
Rotenone	18.6 ± 1.10	<LOQ	–	>46.2
SpinosadA	29.0 ± 1.15	13.6 ± 2.10*	0.47	53.1
SpinosadD	27.2 ± 0.91	14.8 ± 1.06*	0.54	45.6
Spiroxamine	37.8 ± 4.59	17.7 ± 1.31*	0.47	53.1
Tebuconazole	<LOQ	<LOQ	–	–
Tebufenpyrad	31.6 ± 3.48	19.9 ± 1.86*	0.63	37.0
Terbuthylazine	35.3 ± 0.40	23.9 ± 2.98*	0.68	32.1
Tetraconazole	39.5 ± 4.30	15.7 ± 1.88*	0.40	60.1
Thiabendazole	26.2 ± 6.52	12.8 ± 2.49*	0.49	51.3
Thiacloprid	28.1 ± 3.11	14.6 ± 1.85*	0.52	48.2
Triadimefon	37.6 ± 0.61	27.1 ± 3.25*	0.72	27.9
Triadimenol	38.8 ± 3.35	18.9 ± 2.42*	0.49	51.2
Tricyclazole	26.6 ± 2.21	13.9 ± 1.01*	0.52	47.6
Triflumuron	32.0 ± 5.43	11.7 ± 1.52*	0.37	63.3
Zoxamide	27.5 ± 0.13	23.0 ± 1.87*	0.84	16.2
Fludioxonil	24.1 ± 1.64	24.1 ± 1.96*	1.00	0.21

**Legend:** DMST- *N*,*N*-dimethyl-N′-p-tolysulphamide; EPN- O-ethyl O-4-nitrophenyl phosphonothiate.

The asterisk (*) means a significant difference (*p* < 0.05) between the concentration of unprocessed and washed samples, using the Student's t-test.

For the contamination level of 20 μg/kg, from the 121 pesticides included in the mixture used for contamination, only 97 could be quantified. From these, only 40 pesticides presented values higher than the LOQ, including azoxystrobin (strobilurin), buprofezin (pyrimidine) and diniconazole (triazol). The mean concentration of the studied pesticides was in the range of 5.39 and 16.66 μg/kg.

Just as at the concentration of 20 μg/kg, at the concentration of 50 μg/kg of the 121 pesticides validated, only 97 pesticides could be quantified. However, some pesticides in the unprocessed samples were lower than the LOQ, such as carboxin (carboxanilide), coumaphos (organophosphate), etrimfos (organophosphate), fonofos (organophosphate), isoprothiolane (dithiolane), metaflumizone (semicarbazone), fhosphamidon (organophosphate), pirimiphos-ethly (organophosphate) and tebuconazole (triazole).

The mean concentration of the studied pesticides was in the range of 11.27–41.73 μg/kg. As mentioned before, the rice was dried in the sun until completely dry.

#### 3.2.2 Effects of washing

For the concentration of 20 μg/kg of the 97 pesticides, it was only possible to calculate the percentage reduction for 22 pesticides. The percentage reduction after washing ranged from 0.53 to 30.4 %. The highest percentage reduction was 30.4 % for fenpropimorph (morpholine), followed by 27.2 % for spiroxamine (spiroketalamide). As mentioned above, some pesticides are below the LOQ, so it was not possible to quantify their reduction (section 3.2.1.). Although the reduction of concentration of 22 residues of pesticides, only seven residues of pesticides showed a significant reduction (*p* < 0.05), namely diazinon, epoxiconazole, fenpropimorph, flutriafol, mepanipyrim, spinosad A and spiroxamine. This suggests that, for these pesticides, washing is a significant mitigation strategy to reduce human exposure through rice consumption. However, for most of the pesticides tested at this concentration, the reductions were less pronounced, with some residues remaining below the LOQ, making it difficult to assess their overall behavior during washing. Therefore, the corresponding LOQ of the pesticide was used to calculate the minimal percentage reduction (Table 2, Table 3).

For the concentration of 50 μg/kg of the 97 pesticides, it was only possible to calculate the percentage reduction for 86 pesticides. The percentage reduction after washing ranged from 0.21 to 73 %. The highest reduction was 73 % for pirimiphos-methyl (organophosphate), followed by 65.9 % for fenamidone (imidazole), 65.8 % for cadusafos (organophosphate) and 65.4 % for propiconazole (azole). The pesticides that are below the LOQ after washing are EPN (organophosphate), fenamiphos sulfoxide (organophosphate), fenthion oxon (organophosphate), and fenthion oxon sulfoxide (organophosphate).

It is important to note that significant reductions (*p* < 0.05) were observed for 78 of the 97 pesticides tested, indicating that washing had a greater overall impact on this higher level of contamination. This may be due to a higher initial residue load, which increases the likelihood of removal, or to differences in the behavior of pesticides at different concentrations. Interestingly, some pesticides, such as diazinon (organophosphate), indoxacarb (oxadiacin), zoxamide (benzamide), and fludioxonil (phenylpyrrole), were not significantly reduced by washing at any of the concentrations.

The results also suggest that the initial concentration of pesticide residues plays a crucial role in the efficacy of washing. At lower concentrations (20 μg/kg), more than half of the pesticides were below the LOQ, meaning they were either present in minimal amounts or removed to undetectable levels. In contrast, at 50 μg/kg, fewer pesticides were below the LOQ, but a larger proportion showed statistically significant reductions. This highlights the importance of considering residue concentration when evaluating the effectiveness of decontamination processes. Furthermore, the difference in reduction efficiency between the two concentrations could inform regulatory guidelines regarding maximum allowable pesticide residues in rice and the importance of pre-consumption washing as a risk mitigation strategy.

**Fig. 1:**
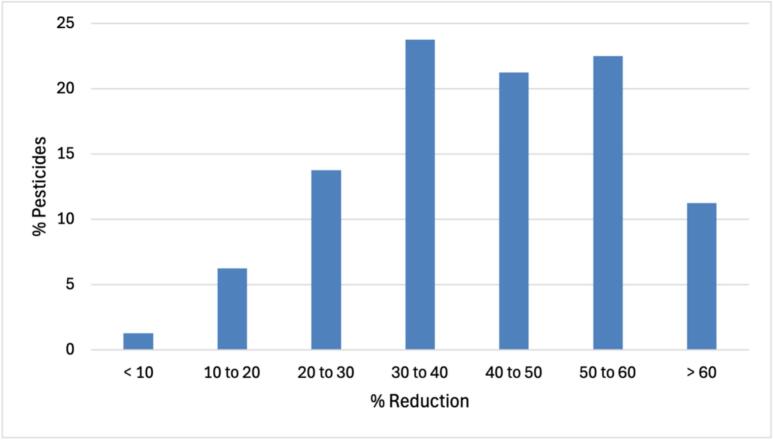
Reduction of pesticides in a contaminated rice samples (at 50 μg/kg) after washing with mineral water.

More than 40 % of the pesticides presented a reduction between 40 and 60 %, and more than 10 % of pesticides presented a reduction higher than 60 % just after washing with mineral water (Fig. 1). The mode of action plays an important role in residue removal from the products through washing processes. Contact pesticides are often used on commodity surfaces, where they are readily removed by washing procedures as they do not typically penetrate the product. Like carbaryl, cadusafos, and triflumuron. Systemic pesticides, on the other hand, penetrate the product. The plant's vascular system allows pesticide sprays to be absorbed by the leaves and stems and then transported to different parts of the plant. Therefore, using washing techniques to get rid of systemic pesticides is extremely difficult or perhaps impossible. Some examples of systemic pesticides are bupirimate, propyzamide and thiacloprid (Gıda ve Yem Kontrol Merkez Araştırma Enstitüsü Müdürlüğü tarafından yayınlanmıştır et al., 2023).

Comparing the results of a systemic pesticide (propyzamide) and a non-systemic pesticide (cadusafos), cadusafos achieves greater reductions. In long-grain rice, washing with water reduced 65.8 %, while propyzamide only reduced 39.1 % when washing with water, proving that it is more difficult to eliminate systemic pesticides than contact pesticides, however it is possible to reduce the presence of systemic pesticides.

**Fig. 2:**
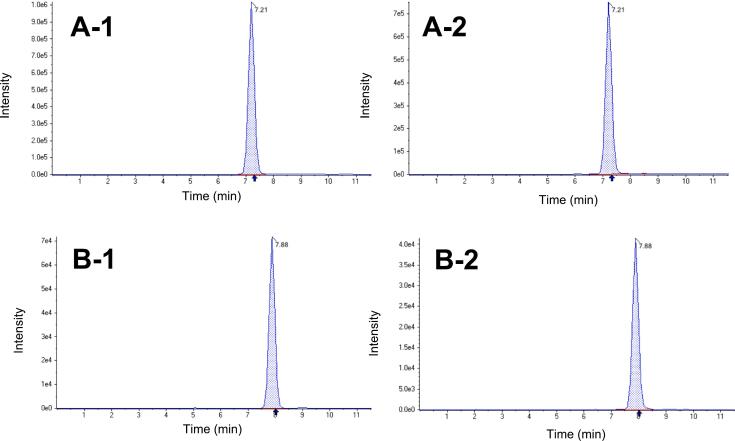
Chromatograms (intensity vs. time (min)) of different samples: unprocessed (1) and washed (2) for (A)- Azoxystrobin and (B)- Bixafen as representative pesticides residues.

Fig. 2 shows the difference in pesticide concentration for the different samples: unprocessed (1) and washed (2), for azoxystrobin and bixafen, two representative pesticide residues.

## 4 Concluding remarks

Pesticide residues in food are a significant concern due to their association with adverse human health effects. Consequently, developing straightforward and cost-effective analytical methodologies is crucial to enhance food safety and mitigate potential harmful contaminants such as pesticide residues, particularly in staple foods like rice.

Therefore, with this work it is possible to quantify by HPLC-MS/MS a very wide range of different types of pesticides (121) at very low concentrations and apply this method to evaluate the effect of washing on long grain rice samples.

With this study, it can be concluded that washing rice influences reducing pesticides, and with washing there were many pesticides that reduced by more than 50 %. This work has some limitations, as data processing takes too long to analyze each pesticide individually. However, it always be effective because it has been shown that washing rice with just water reduces the presence of pesticides.

In the future it would be interesting to continue studying the pesticides residues in another types of rice, such as, basmati rice, brown rice, and *Carolino* rice (short grain). It would be interesting studying the effect of washing with vinegar in the reduction of pesticides residues in contaminated samples, using apple cider vinegar. Moreover, instead of using acetic acid, the effect of citric acid (or directly lemon juice) could also be evaluated. It would be appealing to also evaluate the reduction of these pesticides when cooking the rice. It would also be interesting to carry out the study on other cereals (such as oats and rye), or even on pasta and also on pseudocereals such as amaranth, quinoa and buckwheat.

## Acknowledgements and Funding

The study was funded by project TRACE-RICE—Tracing rice and valorizing side streams along Mediterranean blockchain, grant No. 1934, of the PRIMA Programme, supported under Horizon 2020, the European Union's Framework Programme for Research and Innovation. This research was also funded by PT national funds (FCT/MCTES, Fundação para a Ciência e Tecnologia and Ministério da Ciência, Tecnologia e Ensino Superior) through the grant UIDB/00211/2020. F. Carreiró is grateful for her fellowship in the frame of the TRACE-RICE project.

## CRediT authorship contribution statement

**Filipa Carreiró:** Writing – original draft, Software, Resources, Methodology, Investigation, Formal analysis, Data curation. **Sílvia Cruz Barros:** Writing – original draft, Visualization, Validation, Software, Resources, Methodology, Investigation, Formal analysis, Data curation, Conceptualization. **Carla Brites:** Writing – review & editing, Project administration, Funding acquisition. **Ana Rita Mateus:** Writing – review & editing, Software, Formal analysis. **Fernando Ramos:** Writing – review & editing, Supervision. **Duarte Torres:** Writing – review & editing, Supervision, Investigation. **Ana Sanches Silva:** Writing – review & editing, Supervision, Project administration, Funding acquisition.

## Declaration of competing interest

The authors declare that they have no known competing financial interests or personal relationships that could have appeared to influence the work reported in this paper.

## Figures and Tables

**Table A1:** Parameters for determination of pesticides residues in rice by HPLC-MS/MS in ESI+ mode. Transition 1: Quantification transition; Transition 2: Confirmation transition.

**Pesticides**	**Transitions**	**Precursor ion** **(*m*/*z*** ± **0.5)**	**Product ion** **(m/z** ± **0.5)**	**DP (V)**	**CE (V)**	**CXP (V)**
**TPP**		327	152	106	47	10
Acetamiprid	1	223	126	74	27	4
	2	225	128	61	29	8
Azoxystrobin	1	404	372	46	21	10
	2	404	344	46	35	20
Bixafen	1	414	394	76	21	24
	2	416	396	81	23	16
Bitertanol	1	338	99	51	21	6
	2	338	269	51	13	20
Boscalid	1	343	307	76	29	18
	2	343	140	116	27	6
Bupirimate	1	317	272	61	27	22
	2	317	108	91	41	6
Buprofezin	1	306	201	51	17	14
	2	306	116	56	23	20
Carbaryl	1	202	145	86	17	16
	2	202	127	86	28	14
Carbendazim	1	192	160	51	25	10
	2	192	105	61	51	18
Carbofuran	1	222	165	56	15	22
	2	222	123	56	25	14
Carbofuran-3-hydroxy	1	238	181	82	17	10
	2	238	163	82	21	10
Carboxin	1	236	143	46	23	8
	2	236	87	46	35	14
Chlorantraniliprole	1	484	99	51	21	16
	2	484	286	61	23	6
Chlorfenvinphos	1	359	99	75	41	6
	2	359	155	75	19	8
Chlorpyrifos-methyl	1	324	125	66	25	8
	2	322	125	66	29	6
Clofentezine	1	303	138	56	21	20
	2	303	102	56	59	18
Coumaphos	1	363	227	84	37	12
	2	363	307	84	23	7
Cymoxanil	1	199	128	46	35	14
	2	199	83	46	13	8
Cyproconazole	1	292	70	71	35	14
	2	294	125	16	35	4
Cyprodinil	1	226	91	81	49	16
	2	226	93	81	47	16
Demeton-*S*-methylsulfone	1	263	109	46	21	10
	2	263	169	76	37	20
Diazinon	1	414	394	76	16	6
	2	416	396	81	23	6
Dichlorvos	1	221	109	76	25	4
	2	223	109	66	23	18
Difenoconazole	1	406	251	81	37	4
	2	406	337	41	23	13
Diflubenzuron	1	311	158	81	19	18
	2	311	141	81	43	20
Dimethoate	1	230	199	61	13	8
	2	230	225	61	29	6
Dimethomorph	1	388	301	51	45	10
	2	388	165	51	29	18
Diniconazole	1	326	159	56	39	13
	2	326	70	56	45	13
DMST	1	215	106	100	10	13
	2	215	151	100	5	13
EPN	1	324	296	16	19	6
	2	324	157	16	29	6
Epoxiconazole	1	330	101	36	63	13
	2	330	121	36	27	13
Ethiofencarb	1	226	107	51	23	18
	2	226	164	51	13	10
Ethoprophos	1	243	97	71	41	4
	2	243	131	66	29	8
Etrimfos	1	293	125	64	33	8
	2	293	265	61	21	10
Fenamidone	1	312	65	81	71	4
	2	312	236	41	19	4
Fenamiphos sulfone	1	336	266	81	29	13
	2	336	188	81	41	13
Fenamiphos sulfoxide	1	320	233	86	35	12
	2	320	108	86	59	6
Fenarimol	1	331	268	86	31	10
	2	331	139	96	49	14
Fenitrothion	1	278	125	71	31	6
	2	278	109	71	25	6
Fenoxycarb	1	302	55	96	59	4
	2	302	97	91	35	6
Fenpropathrin	1	350	97	81	43	4
	2	350	125	76	19	6
Fenpropidin	1	274	147	51	37	7
	2	274	117	51	65	7
Fenpropimorph	1	304	147	74	39	7
	2	304	117	11	71	6
Fenpyroximate	1	422	366.1	76	23	6
	2	422	138	76	41	4
Fenthion oxon sulfone	1	295	217	96	27	12
	2	295	104	96	35	6
Fenthion oxon sulfoxide	1	279	264	56	27	18
	2	279	104	56	39	18
Fenthion oxon	1	263	231	66	23	14
	2	263	216	76	33	14
Fenthion sulfone	1	311	125	76	28	8
	2	311	279	76	27	16
Fenthion sulfoxide	1	295	280	76	25	16
	2	295	109	76	45	18
Fluopyram	1	397	145	81	79	8
	2	397	173	81	43	10
Fluquinconazole	1	376	307	56	33	7
	2	376	349	56	25	14
Flutriafol	1	302	123	41	39	13
	2	302	109	41	43	13
Fonofos	1	247	109	61	26	6
	2	247	137	61	15	6
Fosthiazate	1	284	104	11	27	6
	2	284	228	11	15	8
Hexythiazox	1	353	228	66	21	4
	2	353	168	66	37	14
Indoxacarb	1	528	150	26	31	18
	2	528	203	96	51	7
Iprodione	1	330	245	61	21	14
	2	332	247	61	21	14
Iprovalicarb	1	321	119	56	27	8
	2	321	186	56	17	16
Isoprocarb	1	194	95	65	19	14
	2	194	152	65	11	20
Isoprothiolane	1	291	231	51	17	14
	2	291	189	51	31	12
Isoproturon	1	207	72	61	35	12
	2	207	165	61	21	10
Kresoxim-methyl	1	314	267	75	5	13
	2	314	222	75	9	13
Linuron	1	249	160	61	25	18
	2	249	182	61	21	22
Lufenuron	1	511	158	79	27	4
	2	511	141	79	67	4
Malaoxon	1	315	99	71	31	4
	2	315	127	71	17	6
Malathion	1	331	127	64	17	4
	2	331	285	64	13	4
Mepanipyrim	1	224	106	66	35	7
	2	224	77	66	49	7
Metaflumizone	1	507	178	86	35	10
	2	507	89	86	123	4
Metalaxyl	1	280	192	61	25	10
	2	280	220	61	21	12
Metalaxyl-M	1	280	220	46	19	13
	2	280	160	46	31	13
Metazachlor	1	278	210	41	15	4
	2	278	134	41	29	8
Metconazole	1	320	70	76	45	7
	2	320	125	81	49	6
Methiocarb	1	226	169	101	13	20
	2	226	121	101	25	18
Methomyl	1	163	88	46	13	4
	2	163	106	46	15	18
Metobromuron	1	261	172	76	25	6.5
	2	261	148	76	21	4
Metribuzin	1	215	187	31	25	13
	2	215	84.1	31	29	13
Mevinphos	1	225	127	69	19	4
	2	225	193	69	13	4
Monocrotophos	1	224	127	86	21	6
	2	224	193	86	13	14
Oxadixyl	1	279	219	66	15	4
	2	279	132	66	41	4
Paclobutrazole	1	294	70	71	39	4
	2	294	89	71	81	4
Paraoxon-ethyl	1	276	220	61	19	6
	2	276	248	61	15	16
Paraoxon-methyl	1	248	202	96	25	7
	2	248	90	66	37	6
Parathion	1	292	236	81	21	6.5
	2	292	264	81	15	13
Parathion-methyl	1	264	125	76	25	7
	2	264	109	120	21	13
Penconazole	1	284	70	61	49	12
	2	284	159	61	47	14
Pencycuron	1	329	125	71	33	8
	2	329	218	71	23	14
Pendimethalin	1	282	212	41	17	12
	2	282	194	41	27	12
Phentoate	1	321	79	31	51	6
	2	321	247	16	13	10
Phosalone	1	368	182	66	19	14
	2	368	111	51	51	18
Phosmet	1	318.1	160	56	21	12
	2	318.1	77	56	69	13
Phosphamidon	1	300	127	66	27	6
	2	300	227	66	19	14
Phoxim	1	299	77	56	47	12
	2	299	129	56	17	12
Pirimiphos-ethyl	1	334	198	85	29	8
	2	334	182	85	31	8
Pirimiphos-methyl	1	306	108	71	43	6
	2	306	67	71	65	6
Prochloraz	1	376	308	16	17	13
	2	376	266	16	23	13
Profenofos	1	373	303	115	17	13
	2	375	305	115	17	13
Propiconazole	1	342	159	46	37	13
	2	342	89	86	99	4
Propoxur	1	210	168	58	11	24
	2	210	93	58	33	7
Propyzamide	1	256	173	56	31	8
	2	256	190	56	21	10
Prothioconazole-desthio	1	312	70	76	61	2
	2	312	125	76	49	6
Pyrazophos	1	374	222	81	29	6
	2	374	194	11	43	7
Pyrimethanil	1	200	107	71	35	8
	2	200	82	71	37	14
Quinoxyfen	1	308	197	61	43	7
	2	308	162	61	57	7
Rotenone	1	395	213	81	33	12
	2	395	192	91	31	12
SpinosadA	1	732.5	142	51	37	13
	2	732.5	98	51	75	13
SpinosadD	1	746.5	142	66	39	13
	2	746.5	98	66	79	13
Spiroxamine	1	298	144	41	27	10
	2	298	100	41	41	6
Tebuconazole	1	308	70	21	39	13
	2	308	125	21	47	13
Tebufenpyrad	1	334	117	86	57	6
	2	334	145	86	38	8
Terbuthylazine	1	232	176	81	25	7
	2	230	104	81	45	6
Tetraconazole	1	372	159	81	39	14
	2	374	161	76	35	8
Thiabendazole	1	202	175	79	35	4
	2	202	131	81	47	10
Thiacloprid	1	253	126	99	29	4
	2	255	128	76	31	6
Thidiocarb	1	355	88	31	19	12
	2	355	108	61	21	20
Triadimefon	1	294	225	61	19	14
	2	296	199	36	21	12
Triadimenol	1	296	70	41	37	12
	2	296	227	11	15	13
Tricyclazole	1	190	163	89	31	7
	2	190	136	91	36	7
Triflumuron	1	359	156	21	21	18
	2	359	139	21	45	18
Zoxamide	1	336	187	46	31	24
	2	336	159	46	55	20

**Legend:** DP- Declustering potential (V); CE- Collision energy (V); CXP- Collision cell exist potential (V); DMST- *N*,*N*-dimethyl-N′-p-tolysulphamide; EPN- O-ethyl O-4-nitrophenyl phosphonothiate

**Table A2:** Parameters for determination of pesticides residues in rice by HPLC-MS/MS in ESI- mode. Transition 1: Quantification transition; Transition 2: Confirmation transition.

**Pesticide**	**Transitions**	**Percursor ion** **(m/z** ± **0.5)**	**Product ion** **(m/z** ± **0.5)**	**DP (V)**	**CE (V)**	**CXP (V)**
DNC		301	137	−120	−16	−15
Fludioxonil	1	247	180	−65	−40	−9
	2	247	126	−65	−42	−7

**Legend:** DP- Declustering potential (V); CE- Collision energy (V); CXP- Collision cell exist potential (V)

## Data Availability

The data that has been used is confidential.
